# Atovaquone Impairs Growth of *Aspergillus* and *Fusarium* Keratitis Isolates by Modulating Mitochondrial Function and Zinc Homeostasis

**DOI:** 10.1167/iovs.17-22585

**Published:** 2018-03

**Authors:** Heather L. Clark, Martin S. Minns, Yan Sun, Tristan de Jesus, Mahmoud G. Ghannoum, Eric Pearlman

**Affiliations:** 1 Department of Ophthalmology and Visual Sciences, Case Western Reserve University, Cleveland, Ohio, United States; 2 Department of Ophthalmology, University of California Irvine, Irvine, California, United States; 3 Department of Physiology and Biophysics, University of California Irvine, Irvine, California, United States; 4 Department of Dermatology, Case Western Reserve University, Cleveland, Ohio, United States

**Keywords:** *Aspergillus*, *Fusarium*, fungal keratitis, atovaquone

## Abstract

**Purpose:**

*Aspergillus* and *Fusarium* molds cause blinding corneal infections as a consequence of ocular trauma and in association with contact lens wear. As these fungi require zinc for fungal growth, we examined the effect of atovaquone, a ubiquinone analog that disrupts zinc homeostasis, on fungal growth in vitro and in vivo.

**Methods:**

In vitro: *Aspergillus* and *Fusarium* germinating conidia were incubated overnight with atovaquone, and hyphal growth was measured by fluorimetry. In vivo: C57BL/6 mouse corneas were infected with *Aspergillus* or *Fusarium* conidia. Atovaquone was added topically and corneal opacification and fungal growth were quantified.

**Results:**

Atovaquone has antifungal activity against *Aspergillus* and *Fusarium* clinical isolates, with *Fusarium* species being more sensitive to atovaquone than *Aspergillus* species. Atovaquone also reduced labile intracellular zinc levels and increased the sensitivity of *Aspergillus* to metal shock. Atovaquone reduced vacuolar acidification, which regulates storage of intracellular free zinc, and also acted synergistically with voriconazole and itraconazole to kill hyphae. Furthermore, mitochondrial potential and ATP production were reduced in both *Aspergillus* and *Fusarium* following atovaquone treatment. Finally, topical application of atovaquone to the ocular surface significantly inhibited fungal growth and corneal opacification in murine models of fungal keratitis.

**Conclusions:**

These studies demonstrate that atovaquone has pronounced in vitro and in vivo antifungal activity against filamentous fungi by disrupting both metal homeostasis and mitochondrial function, and therefore has potential as a novel antifungal agent.

*Aspergillus* and *Fusarium* are filamentous molds that are ubiquitous in the environment; however, they can cause severe pulmonary, dermatologic, and systemic infections in patients with genetic or induced immune deficiencies, which frequently results in death from uncontrolled fungal growth.^1–4^ *Fusarium* species are also an important cause of contact lens–related corneal infections in immune-competent individuals in the industrialized world, and *Fusarium* and *Aspergillus* corneal ulcers are common in developing countries following ocular injury and penetration of spores to the corneal stroma.^5–7^ Fungal keratitis is painful, blinding, and frequently requires corneal transplantation. Also, current topical and systemic antifungal agents have limited efficacy, and there are several reports of resistance to commonly used drugs, such as azoles.^8–11^ Furthermore, many antifungal agents have severe side effects, including infusion reactions and nephrotoxicity.^12,13^ Overall, there is an unmet need for new drugs that target filamentous fungi, and that are safe and effective.^14,15^

We reported that the antimicrobial peptide calprotectin (S100A8/A9) produced by neutrophils inhibits *Aspergillus fumigatus* hyphal growth by successfully competing for free zinc and manganese, and that recombinant calprotectin inhibits experimental *Aspergillus* corneal infections.^16^ These findings demonstrate that zinc uptake is a potential target for antifungal therapy.

In the current study, we examined the role of the ubiquinone analog atovaquone to target zinc homeostasis in *Aspergillus* keratitis. Atovaquone is a hydroxy-1,4-naphthoquinone that binds to cytochrome b and interferes with the electron transport chain and respiration in the protozoan parasites *Plasmodium*, *Toxoplasma*, *Babesia,* and *Leishmania*.^17–22^ Atovaquone is approved by the Food and Drug Administration for treatment of pneumonia caused by the pathogenic yeast *Pneumocystis carinii* and for malaria in combination therapy with proguanil hydrochloride (Malarone).^23^

Atovaquone also disrupts zinc homeostasis in *Candida albicans* and *Saccharomyces cerevisiae* yeasts^24^; however, there are to date no reports on the effect of atovaquone on pathogenic molds.

Here, we demonstrate that atovaquone functions as an effective antifungal agent by disrupting both mitochondrial function and intracellular zinc storage in *Aspergillus* and *Fusarium* species, and that topical application inhibits hyphal growth in infected corneas.

## Materials and Methods

### Fungal Strains and Growth Conditions

The sources of *Fusarium* and *Aspergillus* clinical isolates are listed in the Table above. The ATP Binding Cassette (ABC) transporter mutant *HspA-Cdr1A* and parent strains *A. fumigatus* were generously provided by Scott Moye-Rowley (University of Iowa). *Fusarium* strains were cultured at 30°C on Sabouraud dextrose agar (SDA), whereas *Aspergillus* strains were cultured on SDA at 37°C for 48 to 72 hours until conidiophores were generated. Conidia (spores) were harvested by scraping the plates, re-suspending conidia in sterile PBS and filtering through sterile gauze to remove hyphae.

**Table:**
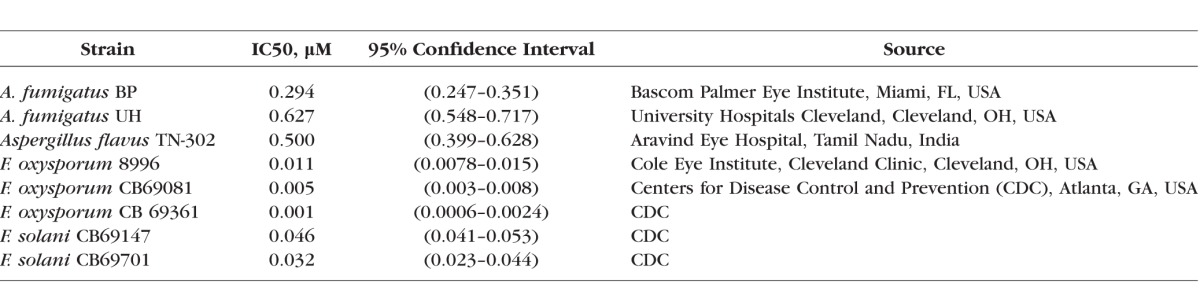
Inhibitory Concentrations of Atovaquone on Keratitis Clinical Isolates

### In Vitro Hyphal Killing Assays

Conidia were plated at 5 × 10^3^/mL in 200 μL of Sabouraud Dextrose broth (SDB; Sigma-Aldrich, St. Louis, MO, USA). in black-walled 96-well plates with clear bottoms (Corning Inc., Corning, NY, USA), and incubated until germination (6 hours for *Aspergillus*, and 24 hours for the slower-growing *Fusarium*) as described.^25^ For all experiments, *Fusarium* strains were cultured at 30°C, whereas *Aspergillus* strains were cultured at 37°C.

Atovaquone was purchased from Sigma-Aldrich and stock solution (25 mM) was prepared in dimethyl sulfoxide (DMSO) and stored at −20°C. Amphotericin B solution was purchased from Sigma-Aldrich, itraconazole and voriconazole were purchased from Cayman Chemical (Ann Arbor, MI, USA), and 20 μg/mL stock solution was prepared in DMSO and stored at −20°C.

Germinating conidia were incubated with 2-fold serial dilutions of atovaquone, Amphotericin B, itraconazole, or voriconazole (or DMSO control) in RPMI 1640. Incubation times were 18 hours for *Aspergillus* strains or 48 hours for *Fusarium* strains, which is when hyphae in RPMI alone reached maximal growth. Supernatants were then removed and the chitin-binding stain Calcofluor white (Sigma-Aldrich) was added at 50 μL per well for 10 minutes at room temperature (RT). Wells were washed three times in double-distilled H_2_0 and raw fluorescent units (RFUs) were quantified using a Biotek (Winooski, VT, USA) Cytation 5 at 360/440 nM, as we described.^16^ Images were acquired on the Cytation 5 using a 4′,6-diamidino-2-phenylindole filter. Data are presented as the percentage of Calcofluor white fluorescence in RPMI with no drugs added (RFU + drugs/RFU − drugs × 100).

### Mitochondrial Potential and ATP Measurement

Hyphae were incubated 2 hours with atovaquone, mitochondria inhibitor carbonyl cyanide m-chlorophenyl hydrazone (CCCP) or DMSO in RPMI 1640. RPMI was then removed and fresh RPMI 1640 (no phenol red) containing JC1 (2 μM) probe was added for 15 minutes, and fluorescence was quantified per the manufacturer's directions (MitoProbe JC1 Assay Kit; ThermoFisher Scientific, Waltham, MA, USA). JC-1 dye accumulates in healthy mitochondria (intact mitochondrial potential), indicated by a fluorescence emission shift from green (∼529 nm) to red (∼590 nm); consequently, mitochondrial depolarization is indicated by a decrease in the red/green fluorescence intensity ratio.

For ATP measurements, hyphae were treated with atovaquone or medium alone in 200 μL RPMI 1640 for 2 hours; 175 μL media was removed and 25 μL BacTiter-Glo Reagent (Promega, Madison, WI, USA) was added to the remaining 25 μL culture media and incubated for 10 minutes at RT. BacTiter-Glo Reagent generates a luminescent signal proportional to the amount of ATP in a sample using a proprietary luciferase reagent (Promega). Luminescence was measured using the Cytation5 reader.

### Fungal Viability Assay

Hyphae were treated with atovaquone or amphotericin B in RPMI plus 0.25 μM SYTOX green extracellular nucleic acid stain (ThermoFisher Scientific) where SYTOX reactivity is only detected in dead cells that have permeable membranes. Fluorescence (RFU) was recorded at 504/523 nm after incubation for 2 hours for *Aspergillus* or 8 hours for *Fusarium*.

### Metal Shock and Zinc Measurements and Imaging

*Aspergillus* and *Fusarium* hyphae were incubated 2 hours with atovaquone, and the zinc chelator TPEN (Sigma-Aldrich) was added at indicated concentrations. To quantify total zinc in the hyphae, 2 μM Zinbo-5 (Santa Cruz Biotechnology, Dallas, TX, USA) in PBS or 25 μM Zinquin (Santa Cruz Biotechnology) in RPMI 1640 were added to wells for 15 minutes. Plates were washed, and fluorescence was read at 358/463 for Zinbo-5 or at 368/490 for Zinquin. For metal shock experiments in which high levels of metals are added to the cultures, conidia were grown to germination in SDB, washed, and RPMI +/− atovaquone +/− ZnSO_4_, MnSO_4_, or CuSO_4_ (Sigma-Aldrich) was added. Hyphae were incubated 18 hours for *Aspergillus* or 48 hours for *Fusarium*. Fluorescence was quantified using Calcofluor white, as described above.

### Acidic Vacuole Quantification

To quantify total acidified vacuoles, hyphae were incubated 2 hours with atovaquone, growth medium was removed, and 200 μL RPMI 1640 with 1 μM Lysosensor Green DND-189 (ThermoFisher Scientific) was added and incubated for 30 minutes. The growth medium was removed and fresh RPMI 1640 was added to each well. Fluorescence was read at 443/505.

### Cytotoxicity Assay

Telomerase immortalized human corneal epithelial cells were plated in KGM Gold media (Lonza, Anaheim, CA, USA) and grown to confluency. Cells were incubated with atovaquone or DMSO vehicle for 24 hours, and cell death was measured by release of lactose dehydrogenase (LDH) into the media. LDH was quantified using CytoTox nonradioactive toxicity assay according to the manufacturer's directions (Promega). Cells were incubated with lysis buffer to measure total LDH, and cytotoxicity was measured as a percentage of total LDH.

### Murine Model of Corneal Infection

Six- to 8-week old male and female C57BL/6 mice were purchased from the Jackson Laboratories (Bar Harbor, ME, USA). *A. fumigatus* and *Fusarium* conidia (50,000 in 2 μL PBS) were injected into the corneal stroma using a 33G Hamilton syringe as we described.^16,26,27^ Atovaquone was diluted in a proprietary eye drop formulation provided by Alcon (Ft. Worth, TX, USA), and drug or vehicle only was dropped onto the ocular surface at 0, 2, and 6 hours postinfection (pi). After 24 hours, mice were imaged using a stereomicroscope. Corneal opacification was measured by image analysis using MetaMorph software (MetaMorph Inc., Nashville, TN, USA) as we previously described.^26^ To measure colony forming units (CFUs), whole eyes were homogenized in sterile PBS using a Mixer Mill MM300 (Retsch, Haan, Germany), serial dilutions were plated on SDA, and CFUs were counted manually.

All animal experiments were approved by the Institutional Animal Care and Use Committee at Case Western Reserve University, and adhered to the ARVO Statement for the Use of Animals in Ophthalmic and Vision Research.

### Statistical Analysis

All in vitro experiments were performed at least three times with three or more replicate wells for each condition. Corneal infection experiments had 5 mice per group, and were repeated twice. GraphPad Prism software (GraphPad, La Jolla, CA, USA) was used for all statistical analysis, which were performed on experimental replicates. Significance was determined using either a Student's *t*-test or ANOVA with Tukey post-analysis. Half maximal inhibitory concentration (IC50) values were calculated using the log(inhibitor) versus response equation, where 100% fungal mass indicates hyphal growth in media alone.

## Results

### Atovaquone Inhibits Growth of *Aspergillus* Clinical Isolates

**Figure 1:**
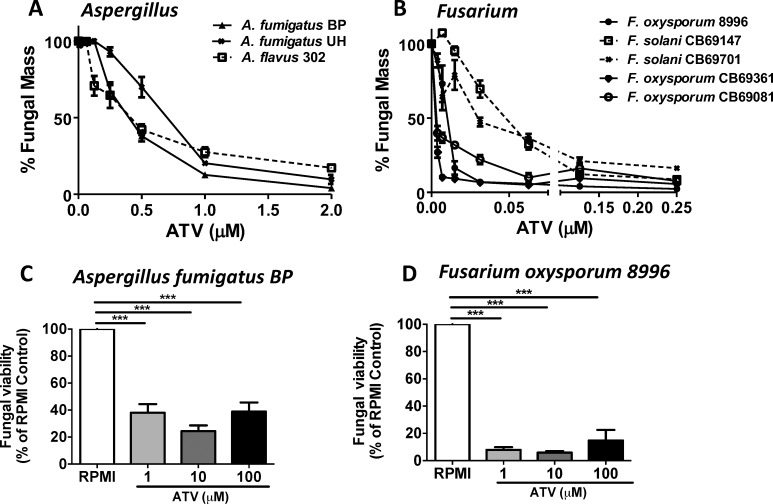
Fungicidal activity of atovaquone on hyphal growth of clinical isolates. (A, B) Aspergillus and Fusarium clinical isolates were incubated in RPMI + increasing doses of atovaquone (ATV), and fungal mass was measured by Calcofluor white chitin stain at 18 hours for A. fumigatus or 48 hours for F. oxysporum. (C, D) A. fumigatus BP or F. oxysporum 8996 were incubated in RPMI +/− ATV in the presence of SYTOX Green, which measures extracellular nucleic acids. Fluorescence was measured after 2 hours, and percent viability was calculated as (fluorescence in RPMI only/fluorescence + ATV) × 100. Experiments were performed at least three times with three or more replicate wells for each condition. *P < 0.05; **P < 0.01; ***P < 0.001.

To determine whether atovaquone can inhibit growth of *Aspergillus* and *Fusarium* molds, conidia from *Aspergillus* and *Fusarium* clinical isolates from fungal keratitis patients were grown to the hyphal stages as described in Methods, incubated with atovaquone, and growth was quantified using Calcofluor white chitin stain. Both *Aspergillus* and *Fusarium* hyphae incubated with atovaquone exhibited significantly impaired hyphal growth compared with growth in RPMI media alone (Figs. 1A, 1B). This concentration range was similar to that of other currently used polyene and azole antifungal agents, including voriconazole and amphotericin B (Supplementary Fig. S1A). *Fusarium oxysporum* and *Fusarium solani* clinical isolates were 10- to 20-fold more sensitive to inhibition than *Aspergillus* (IC50 0.3–0.6 μM for *Aspergillus*, 0.001 to 0.06 μM for *Fusarium* [Table]).

To differentiate between cell death and growth inhibition, *A. fumigatus* BP and *F. oxysporum* 8996 hyphae were incubated with atovaquone, and cell death was measured using the SYTOX green nucleic acid stain, which only binds to DNA of dead, permeable cells. We found significantly less *Aspergillus* and *Fusarium* viability (more SYTOX staining) following incubation with atovaquone compared with controls (Figs. 1C, 1D), indicating that atovaquone actively kills *Aspergillus* and *Fusarium* hyphae.

### Atovaquone Is Not Cytotoxic to Human Corneal Epithelial Cells

To examine if atovaquone is cytotoxic to mammalian cells, we incubated human corneal epithelial cells with increasing concentrations of atovaquone for 24 hours, and measured cell death by release of LDH into the culture medium compared with LDH in cells treated with lysis buffer (maximum lysis). These are the human HCE-T telomerase immortalized corneal epithelial cells generated by Robertson et al.^28^ and which we have used extensively.^29,30^

**Figure 2:**
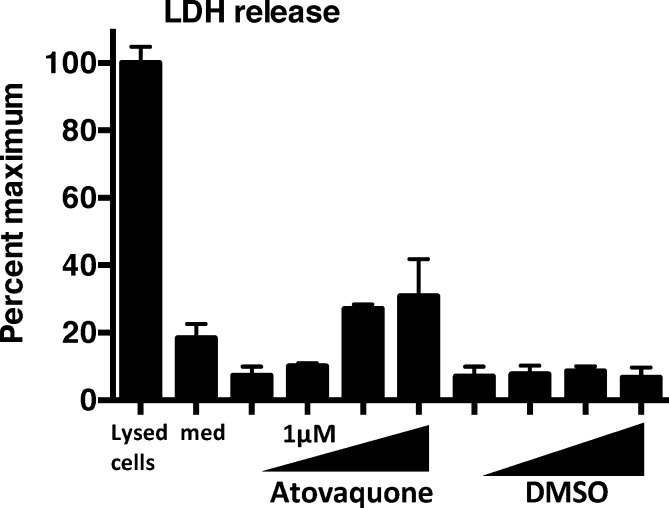
Effect of atovaquone on human corneal epithelial cell viability. LDH release into the culture media compared with Triton X-100 treated cells (100% lysed cells) 24 hours after incubation with atovaquone/DMSO or with DMSO alone Med: culture medium alone. Serial dilutions from 100 nM to 100 μM to atovaquone. Error bars represent six technical replicates. These data are representative of two repeat experiments.

As shown in Figure 2, 10 μM and 100 μM atovaquone showed <20% LDH maximum release. Given that 1 μM atovaquone can inhibit *Aspergillus* and *Fusarium* growth, these data indicate that at the concentrations used, atovaquone has minimal cytotoxic activity on human corneal epithelial cells.

### Atovaquone Reduces Intracellular Zinc Homeostasis in *Aspergillus*

Atovaquone was reported to reduce zinc levels in *C. albicans* yeast, and prior studies show that zinc is critical for *Aspergillus* growth and virulence.^24^ To examine the effect of atovaquone on intracellular zinc levels, hyphae were grown to confluence, treated with atovaquone or the zinc chelator TPEN for 2 hours, and intracellular labile zinc was measured using Zinbo-5 and Zinquin fluorescent probes.

**Figure 3:**
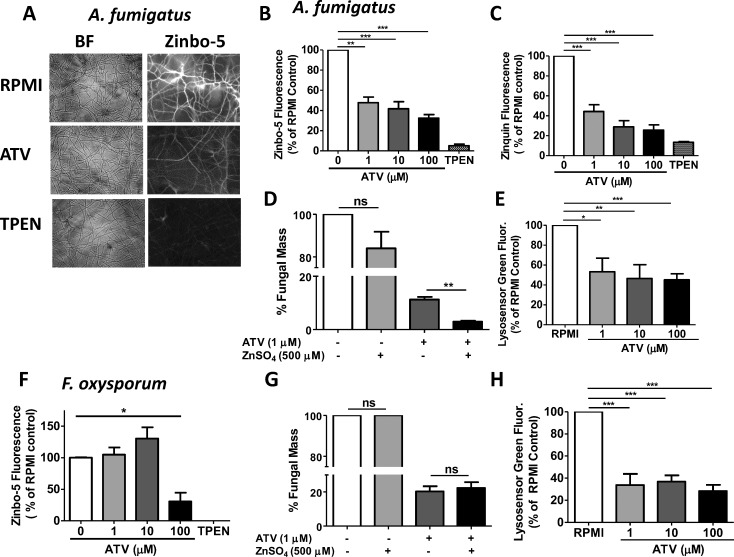
Atovaquone reduces labile intracellular Zn, increases metal toxicity, and disrupts vacuolar acidification. A. fumigatus BP hyphae (A–E) and F. oxysporum hyphae (F–H) were incubated in RPMI +/− ATV or Zn chelator TPEN in the presence of fluorescent labile Zn probe Zinbo-5 for 2 hours. (A) Representative brightfield (BF) and florescence images (×200). (B, C) Zinc content of A. fumigatus using Zn probe Zinbo-5 (B) or Zinquin (C). Fluorescence was normalized to RPMI alone (100%). (D) A. fumigatus hyphal mass following incubation with ATV and ZnSO_4_ (500 μM) measured by Calcofluor white. % Fungal mass = (Growth in ATV + metal/Growth in RPMI + metal only) × 100. (E) A. fumigatus lysosomal activity measured by Lysosensor Green DND-189. (F–H) F. oxysporum 8996: Zinc content measured by Zinbo-5 (F); hyphal mass measured by Calcofluor white (G), and lysosomal activity (H). Experiments were performed at least three times with three or more replicate wells for each condition. *P < 0.05; **P < 0.01; ***P < 0.001.

Representative images revealed bright Zinbo-5 staining in untreated *A. fumigatus* hyphae, but not following incubation with atovaquone or TPEN (Fig. 3A). Quantification of Zinbo-5 and Zinquin fluorescence showed significantly less free zinc in *A. fumigatus* following incubation with atovaquone (Figs. 3B, 3C).

To examine whether an exogenous source of zinc would rescue the effect of atovaquone on *Aspergillus,* hyphae were incubated with 1 μM atovaquone (which partially inhibits hyphal growth) together with an excess of ZnSO_4_. However, rather than restoring growth, the presence of excess zinc further inhibited *A. fumigatus* hyphal growth (Fig. 3D), indicating that atovaquone increases hyphal susceptibility to metal toxicity (metal shock).

Despite the essential role for zinc in fungal metabolism, free zinc and other metals can be toxic to cells at higher levels.^31^ Free metals are stored in specialized vacuoles, which require acidification by V-type ATPases to facilitate metal uptake.^32^ To examine the effect of atovaquone on vacuolar acidification, we measured total acidic vacuoles using the pH-sensitive probe Lysosensor green, which has been used to measure zinc containing vacuoles in eukaryotic cells.^33^ *Aspergillus* hyphae treated with atovaquone had significantly lower Lysosensor green fluorescence compared with RPMI controls (Fig. 3E), indicating that the existing vacuoles are either less acidic or that there are fewer acidic compartments in treated organisms.

In contrast to *Aspergillus*, treatment of *F. oxysporum* 8996 hyphae with atovaquone significantly reduced labile zinc levels at 100 μM, but not at lower doses (Fig. 3F). Furthermore, *F. oxysporum* hyphal growth was not impaired in the presence of atovaquone and excess zinc (Fig. 3G). Treatment of *F. oxysporum* hyphae with atovaquone significantly reduced Lysosensor green fluorescence, thereby showing that atovaquone impaired vacuole acidification (Fig. 3H).

Overall, these data indicate that in *Aspergillus*, atovaquone disrupts metal storage in vacuoles by inhibiting vacuolar acidification, which may explain the increased toxic effects of zinc. *F. oxysporum* shows less sensitivity to zinc modulation by atovaquone.

### Aspergillus ABC Transporters Regulate Atovaquone and Azole Sensitivity

ABC transporters in *S. cerevisiae* are essential for zinc storage in yeast, as loss of ABC transporters results in increased sensitivity to metal toxicity.^34^ Furthermore, ABC transporters mediate resistance to azoles,^35^ and atovaquone inhibits ABC transporter activity in mammalian cells.^36^ To examine the role of ABC transporters on atovaquone and azole sensitivity of filamentous fungi, we used an *A. Fumigatus* mutant that overexpresses the ABC transporter Crd1A (abcA), which we predict will be more resistant to drugs that target ABC transporters. Parent and mutant strains were incubated with partially inhibitory doses of atovaquone alone or together with voriconazole or itraconazole, and growth was measured compared with the parent strain.

**Figure 4:**
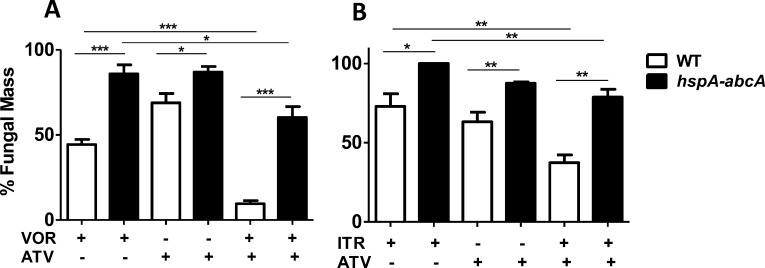
ABC transporter overexpression reduces sensitivity to atovaquone (ATV) and azoles. (A, B) A. fumigatus parent, wild type (WT) strain or AbcA overexpressing strain hsp-AbcA were incubated in RPMI +/− ATV +/− voriconazole (A) or itraconazole (B) for 18 hours and fungal mass was measured using Calcofluor chitin stain. % Fungal mass = (Growth in ATV and/or azole /Growth in RPMI only) × 100. Experiments were performed three times with three replicate wells for each condition. *P < 0.05; **P < 0.01; ***P < 0.001.

As shown in Figure 4A, atovaquone plus voriconazole was more effective than either drug alone in inhibiting growth of the parent *Aspergillus* strain. However, the Crd1 overexpressing mutant *hspA-abcA* strain showed increased resistance to atovaquone either alone or in combination with voriconazole. Similar results were found in separate experiments in which both strains were incubated with atovaquone alone or in combination with itraconazole (Fig. 4B).

These data indicate that *Aspergillus* ABC transporters regulate sensitivity to atovaquone and azoles, and imply that atovaquone potentially disrupts metal storage in *A. fumigatus* by inhibiting ABC transporter activity.

### Atovaquone Disrupts Mitochondrial Potential and ATP Production

The reported mechanism of action of atovaquone on *Plasmodium* is disruption of mitochondrial function.^21,22^ Therefore, we examined the effect of atovaquone on mitochondrial function and ATP production in fungi. *A. fumigatus* and *F. oxysporum* hyphae were incubated 2 hours with 100 μM atovaquone, and mitochondrial potential was measured using a MitoProbe with CCCP as a control mitochondrial inhibitor, as described in Materials and Methods.

**Figure 5:**
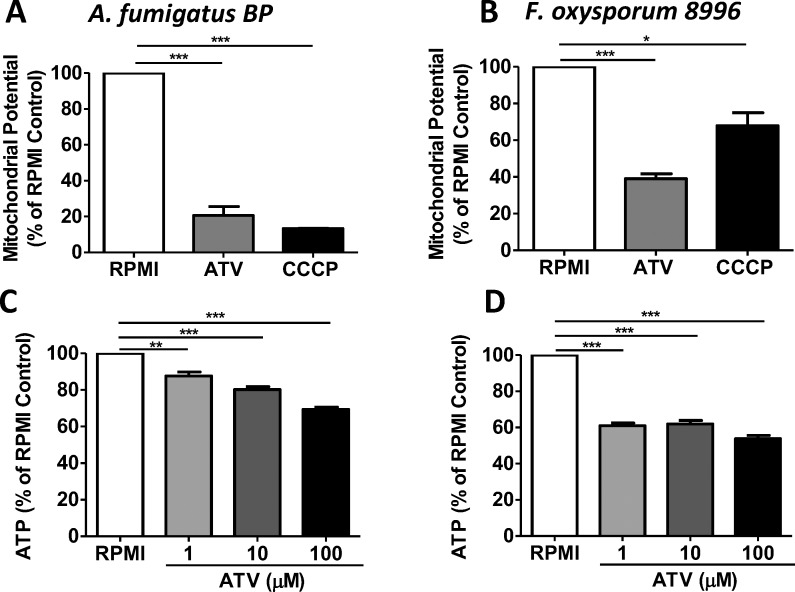
Atovaquone disrupts mitochondrial potential and ATP production. A. fumigatus BP or F. oxysporum 8996 incubated 2 hours in RPMI +/− ATV or CCCP, and mitochondrial potential was measured using the JC-1 probe (A, B). ATP was measured using the luminescent BacTiter-Glo Reagent (C, D). JC-1 and ATP were normalized to RPMI alone (100%). Experiments were performed at least three times with three or more replicate wells for each condition. *P < 0.05; **P < 0.01; ***P < 0.001.

We found that the mitochondrial potential was significantly reduced following incubation with atovaquone (Figs. 5A, 5B). The effect of atovaquone was similar to that of CCCP, a well-characterized inhibitor of mitochondrial potential. Similarly, *A. fumigatus* and *F. oxysporum* ATP production was significantly lower in atovaquone-treated hyphae compared with untreated controls (Figs. 5C, 5D).

Together, these data show that atovaquone also inhibits mitochondrial function and ATP production, resulting not only in inhibition of fungal growth, but also increased hyphal death.

### Atovaquone Inhibits Hyphal Growth and Disease in a Murine Model of Corneal Infection

To examine the effect of atovaquone on *Aspergillus* and *Fusarlium* keratitis, we used a well-characterized model in which corneas are infected intrastromally with *A. fumigatus* or *F. oxysporum* dormant conidia.^26,27^ Atovaquone (50 mM in 2 μL) was applied topically at 0, 2, and 6 hours pi. Mice were euthanized after 24 hours, corneas were imaged for opacification, and CFUs in whole eyes were quantified. Our prior studies showed that the level of corneal opacity corresponds to neutrophil infiltration and live hyphae, that neutrophil recruitment to the cornea peaks at 24 hours, and that there is a strong correlation between CFUs and fungal mass in the cornea.^16,25–27^

**Figure 6:**
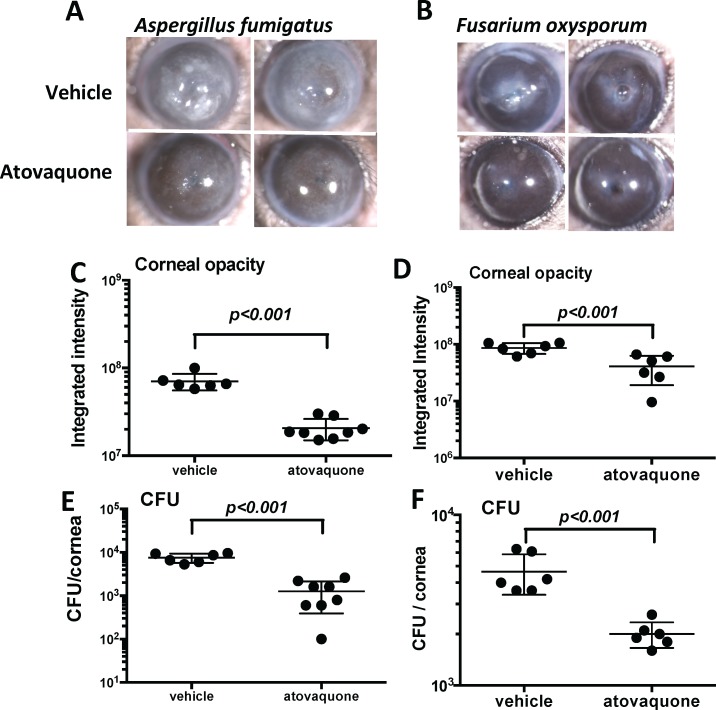
Activity of atovaquone in fungal keratitis. C57BL/6 mice infected intrastromally with A. fumigatus (A, C, E) or F. oxysporum conidia (B, D, F). Mice were treated topically three times with atovaquone at 0, 2, and 6 hours pi. (A, B) Representative images (×20) of corneas at 24 hours pi. (C, D) Quantitative analysis of corneal opacity as described in Materials and Methods. (E, F) CFUs/cornea. Data points represent individual infected corneas. P values were derived by Student's t-test, and experiments were repeated three times with similar results.

Figures 6A–D show significantly less corneal disease in *A. fumigatus*– or *F. oxysporum*–infected corneas treated with atovaquone compared with untreated, infected corneas. CFUs per cornea were also significantly lower in atovaquone-treated corneas (Figs. 6E, 6F). Although *Fusarium* grows slower in vitro, CFU and corneal opacity scores of *F. oxysporum* were similar to that of *A. fumigatus*, indicating that they grow at a similar rate in vivo.

These findings clearly demonstrate that atovaquone is effective in fungal keratitis.

## Discussion

Fungal keratitis caused by filamentous molds is a devastating corneal infection that can lead to pain, permanent blindness, and in severe cases loss of the eye. The limited number of antifungal drugs and increasing resistance to currently used agents indicates that new antifungal drugs are needed. In the current study, we demonstrate that atovaquone is effective in reducing growth of the pathogenic molds *Fusarium* and *Aspergillus* in vitro, and in reducing corneal disease and fungal burden in a murine model of fungal keratitis.

Atovaquone is an analog of ubiquinone/Coenzyme Q, which transfers electrons from dehydrogenases to cytochromes in the electron transport chain.^37^ Atovaquone competitively inhibits coenzyme Q activity and collapses the mitochondrial membrane potential in *Plasmodium*.^37,38^ Although ATP levels are not reduced in atovaquone-treated *Plasmodium*, ATP levels in *P. carinii* yeast were significantly decreased following atovaquone treatment,^39,40^ indicating that atovaquone has more than one mechanism of action, and that the mechanisms may differ among pathogens. Atovaquone inhibits *P. carinii* growth at 0.3 to 3.0 μM, compared with 10 to 100 nM required for *Plasmodium*.^39,40^ In the current study, we demonstrate that atovaquone inhibits the growth of *Aspergillus* in a similar range as *P. carinii* (IC50 0.294–0.5 μM), whereas growth inhibition for *Fusarium* was detected at much lower levels, similar to *Plasmodium* (IC50 0.001–0.046 μM).

We also found that atovaquone reduces both mitochondrial membrane potential and ATP production in *Aspergillus* and *Fusarium*. In addition to directly targeting oxidative phosphorylation through inhibition of electron transport, atovaquone was reported to disrupt zinc homeostasis in an *S. cerevisiae* reporter system and in the pathogenic yeast *C. albicans,* although no mechanism was identified.^24^ Zinc is essential for the catalytic activity of some 300 enzymes, and therefore contributes to growth and survival of microbial pathogens.^41^ We and others^16,42^ reported that blocking zinc uptake with the antimicrobial peptide calprotectin (S100A8/9) inhibits growth of *Aspergillus* and other fungal pathogens, and that zinc acquisition is essential for *Aspergillus* virulence in corneal and pulmonary infections.

Although zinc is required for hyphal growth, divalent cations such as Zn^2+^ are toxic to cells if not effectively stored within the cell.^41^ Mislocalization of metals in the cytosol or other cellular compartments can be detrimental to cells in part by replacing iron in Fe-S centers of enzymes, and by interfering with mitochondrial activity.^41^ Yeast cells have a specific storage vacuole that is required for zinc homeostasis, and which mediates transition metal uptake through Zrc1p in exchange for H^+^ ions. V-type ATPases maintain the vacuole acidification required for nontoxic storage of zinc and other metals,^41^ and *A. fumigatus* ZrcA is the putative orthologue of Zrc1p.^43^ In the current study, we demonstrate using Zinbo-5 and Zinquin dyes that labile (free vacuolar) Zn^2+^ is present in untreated *A. fumigatus* hyphae, but is decreased following incubation with atovaquone. Further, addition of excess Zn^2+^ does not rescue growth, but instead leads to increased fungal killing, suggesting that atovaquone increases the toxicity of Zn^2+^, possibly by blocking proper storage of metals within the cell. Overall*, Fusarium* was found to be less sensitive to zinc toxicity, which may indicate an alternate mechanism of metal regulation in this organism. It is possible that in *Fusarium*, atovaquone primarily affects mitochondrial function rather than zinc homeostasis.

We found that atovaquone reduced acidic compartments in the hyphae, likely vacuoles, thereby supporting a role for atovaquone in inhibiting V-type ATPases. This effect could be due to reduced overall ATP levels, or to a direct inhibitory effect of atovaquone on V-type ATPases. In support of this, V-type ATPase-deficient *S. cerevisiae* yeast and *Aspergillus nidulans* exhibit defects in growth and vacuolar acidification, and are susceptible to metal shock.^44^ V-type ATPases have therefore been proposed as a therapeutic target for antifungal drug development.^44,45^

In addition to V-type ATPases, the ABC G-family transporters of *S. cerevisiae* Pdr18p, Pdr5p, and Pdr15p were shown to interact with zinc transporters Zrtp and Zrc1p, which transport zinc across the plasma membrane and vacuolar membrane, respectively.^34^ Pdr15 and to a lesser extent pdr5 and Pdr18 mutant yeast strains exhibited heightened sensitivity to zinc shock, indicating that ABC transporters are also essential for normal zinc homeostasis, including Zrc1p-mediated zinc storage in vacuoles.^34^ *A. fumigatus* homologs of Zrt1p and Zrc1p, termed ZrfA and ZrcA, have been identified.^43,46^ Recently, it was reported that atovaquone inhibits ABC transporter BCRP/ABCG2 activity in HEK293 cells at 0.23 lM.^36^ However, it has yet to be determined if atovaquone directly affects fungal ABC transporter activity. Enhancing metal sensitivity of pathogens could be a valuable tool due to recent development of metal-based antimicrobial agents, such as QBP, a form of 8-hydroxyquinolone that concentrates copper in the macrophage phagosome and enhances killing of *Cryptococcus neoformans*.^47^

ABC transporters in *Aspergillus* and *Fusarium* are also implicated in azole resistance, including the *A. fumigatus* ABC transporters AbcA and AbcB, which have high sequence similarity to *S. cerevisiae* Pdr5.^35^ Also, *Fusarium graminearum* ABC transporters ABC1, 3 and 4 contribute to azole resistance and virulence in plant hosts.^48^ Given that atovaquone potentially inhibits ABC transporters, it could increase sensitivity to currently used azole drugs if used in combination. Consistent with this possibility, we show in the current study that fungi treated with atovaquone together with either itraconazole or voriconazole have a greater inhibitory effect on fungal growth than either atovaquone or azole alone. Further, the synergistic effect of atovaquone does not occur when an ABC transporter is overexpressed, as in the *hspA-abcA* mutant, indicating that atovaquone may also affect ABC transporter activity. These findings show that atovaquone increases the effectiveness of azoles, and indicates that combination therapy could be an effective treatment, particularly in the case of azole-resistant fungi. Although human cells also possess ABC transporters, atovaquone has a good safety profile and, as noted above, is widely used therapeutically.

Although given before infection, and at higher concentrations than required in vitro, our findings provide a proof of concept that atovaquone can be used to treat filamentous fungal infections, as we demonstrated that treatment of *Aspergillus-* or *Fusarium*-infected corneas with atovaquone reduced fungal burden and corneal opacification. Current antifungal drugs used in keratitis patients, such as natamycin, are given by topical delivery. Although topical delivery to and penetration of drug into the cornea are much less efficient, particularly with a hydrophobic drug such as atovaquone, new delivery methods are under investigation, including cyclodextrin compounds, which form binding pockets for lipophilic drugs to facilitate distribution and release, and have been shown to improve ocular delivery of voriconazole.^49^ These findings will enable topical use at much lower concentrations than used here. Furthermore, new technologies, including contact lens release and ocular “nanowafers,” are also likely to improve ocular drug delivery and increase effectiveness.^50,51^

Atovaquone is currently dosed orally for malaria and *P. carinii* infections, and the effective range of atovaquone against filamentous fungi we demonstrate is well below steady-state plasma concentrations measured in patients (40–82 μM)^23^; therefore, it is possible to reach inhibitory concentrations in plasma in vivo following oral administration. However, it is unclear whether oral drug delivery would result in high enough levels in the cornea. One indication that atovaquone can reach the cornea is that lipophilic drugs, such as atovaquone, when taken orally, can accumulate in the basal corneal epithelium.^52^ Although this is associated with a corneal epithelium defect, symptoms are typically minor and resolve with cessation of treatment. Consistent with those reports, we also show that atovaquone has no cytotoxic effect on the viability of human corneal epithelial cells.

In summary, we show that atovaquone directly kills and inhibits growth of filamentous fungi through at least two mechanisms. Firstly, atovaquone acts on the mitochondria to inhibit electron transport and reduce the mitochondrial potential, resulting in decreased ATP production and cell death. Secondly, atovaquone disrupts metal homeostasis either directly by inhibiting ABC transporters that are required for nontoxic storage of metal, or indirectly through reducing ATP levels, resulting in impaired V-type ATPase-mediated vacuolar acidification and sequestration of zinc. This results in the presence of free zinc in the cytosol, where it disrupts enzyme function and induces oxidative stress.^53^

These studies show that atovaquone is a potential new therapy for filamentous fungal infections when given either alone or together with azoles. Filamentous fungi, especially *Aspergillus* species, also cause opportunistic lung and systemic infections in human immunodeficiency virus–infected and other immune-compromised individuals; therefore, in addition to keratitis patients, atovaquone may be a useful drug for these patients. Furthermore, these studies indicate that fungal metal homeostasis, V-type ATPases, and ABC transporters represent new targets for development of antifungal agents.
